# Peroxymonosulfate enhanced photocatalytic degradation of organic dye by metal-free TpTt-COF under visible light irradiation

**DOI:** 10.1038/s41598-024-58761-w

**Published:** 2024-04-08

**Authors:** Nong Xu, Kaixuan Liu, Qiao Liu, Qing Wang, Anzheng Zhu, Long Fan

**Affiliations:** 1 School of Energy, Materials and Chemical Engineering, Hefei University, Hefei, 230601 People’s Republic of China; 2 State Key Laboratory of Biochemical Engineering, Institute of Process Engineering, Chinese Academy of Science, Beijing, 100190 People’s Republic of China; 3 State Key Laboratory of Materials-Oriented Chemical Engineering, Nanjing Tech University, Nanjing, 211895 People’s Republic of China

**Keywords:** Chemical engineering, Photocatalysis

## Abstract

Recently, the activation of persulfate (PDS) by non-metallic photocatalysts under visible light has attracted significant interest in applications in environmental remediation. This study presents a pioneering investigation into the combined application of the TpTt-COF and PMS for visible light degradation of organic dyes. Synthesized orange TpTt-COF monomers exhibit exceptional crystallinity, a 2D structure, and notable stability in harsh conditions. The broad visible light absorption around a wavelength of 708 nm. The TpTt-COF emerges as a promising candidate for photocatalytic dye degradation. The study addresses high charge recombination in the TpTt-COF, highlighting the crucial role of its electron donor and acceptor for the PMS activation. Comparative analyses against traditional photocatalytic materials, such as the metal-free carbon-based material g-C_3_N_4_ and transition metal-containing TiO_2_, demonstrate TpTt-COF's superior performance, generating diverse free radicals. In simulated experiments, the TpTt-COF's degradation rate surpasses PMS-combined g-C_3_N_4_ by 13.9 times. and 1.6 times higher than the TpTt-COF alone. Remarkably, the TpTt-COF maintains high activity under harsh environments. Investigations into the degradation mechanism and the TpTt-COF's reusability reveal its efficiency and stability. Under visible light, TpTt-COF facilitates efficient electron–hole separation. Combining the TpTt-COF with PMS produces various radicals, ensuring effective separation and a synergistic effect. Radical quenching experiments confirm the pivotal role of O_2_^-^· radicals, while ·OH and SO_4_^-^· radicals intensify the degradation. After five cycles, TpTt-COF maintains an impressive 83.2% degradation efficiency. This study introduces an efficient photocatalytic system mediated by PMS and valuable insights into governing mechanisms for organic pollutant degradation in water environments.

## Introduction

Over the past decades, water pollution has become increasingly severe due to the expansion of urbanization and industrialization, combined with population growth^1^. A substantial portion of this increase is attributed to the significant rise in wastewater containing various organic and inorganic hazards, commonly referred to as recalcitrant pollutants^2^, including antibiotics, dyes, and others. The widespread use of dyes in various industries related to printing and dyeing has significantly contributed to water pollution due to organic toxicity^3^. Representative dyes such as Rhodamine B, Methylene Blue, Methyl Orange, and Congo Red possess challenging abilities such as impacting aquatic ecosystems, affecting crop growth, and being difficult to degrade. When discharged into the water environments, recalcitrant pollutants can seriously harm aquatic organisms and human health^4^. Consequently, the effective removal of organic dyes has become a pressing concern, particularly in developing countries where the demand for dyes is continually growing^5^.

Recently, different methods have been developed for removing dyes from water, including adsorption^6^, biodegradation^7^, chemical oxidation^8^, advanced oxidation processes (AOPs), and so on. Among the proposed methods, AOPs are considered one of the most promising technologies for the complete degradation of various dyes^9^. This technique employs highly effective oxidants for directly breaking down pollutants or utilizing the formation of oxidant-induced radicals to facilitate the process of decomposition^10^. The approach shows considerable potential for breaking down organic matter by generating highly effective and responsive reactive oxidizing species (ROS), such as hydroxyl radicals, superoxide radicals, singlet oxygen, and others. Various oxides, such as hydrogen peroxide (H_2_O_2_), sulfate (SO_4_^2−^), peroxymonosulfate (PMS, HSO_5_^-^), peroxydisulfate (PDS, S_2_O_8_^2−^), and peracetic acid (CH_3_CO_3_H), are activated through different mechanisms to produce ROSs via a sequence of radical chain reactions for the breakdown of pollutants. The resulting ROSs engage in redox reactions with organic matter, ultimately converting it into simple carbon dioxide and water. The sulfate radicals formed by the activation of PMS exhibit higher oxidation potential (2.50–3.10 V) and a longer half-life of 30–40 μs (Eo = 1.90–2.70 V vs. NHE, with a half-life of 20 ns) compared to •OH radicals generated by other AOPs additives. Additionally, they possess characteristics such as high selectivity, environmental friendliness, convenient transport, and low cost. Moreover, the activation of various peroxydisulfates by transition metal ions is a gentle process that does not require additional energy. For Example, Xiao et al.^11^ synthesized Fe-MOF for catalytic activating PMS for the rapid degradation of methylene blue. Although transition metal-activated PMS produces highly oxidizing sulfate free radicals (SO_4_^−∙^) with strong degradation capacity, the metal ions are difficult to recover from high to low valence state, limiting the overall degradation capacity in PMS systems.

Photocatalysis is an efficient and environmentally friendly technology that converts solar energy into chemical activity^12^. Photocatalytic materials including transition metal sulfides^13^ and oxides^14^, biomass-derived carbon^15^, and others. These materials absorb light energy, initiating the transition of electrons within the catalyst from the ground state to an excited state, resulting in the formation of electron–hole pairs. Subsequently, the interaction of electrons and holes with oxygen or water generates reactive oxygen radicals, such as ∙OH and ∙O^2−^. These radicals then engage in mutual interactions with pollutants, facilitating their adsorption onto the catalyst surface and ultimately contributing to the degradation of pollutants. Additionally, photocatalysis can be combined with SR-AOPs to oxidize organic contaminants. Numerous studies demonstrated that, under visible light irradiation, photocatalysts excited electrons and holes to react with PMS, generating SO_4_^-^∙ and SO_5_^-^∙ free radicals. These radicals can attack organic pollutants, breaking them down into smaller fragments. For example, Zhang et al.^9^ utilized an iron-based MOF (MIL-88A) to activate PMS for the visible light catalytic degradation of tetracycline. The Fe-MOF activated PS generated ·OH, O_2_^−·^, SO_4_^−·^, S_2_O_8_^−·^, and other free radicals that were responsible for completely degrading tetracycline after 80 min of visible light illumination. However, these materials often suffer from rapid charge recombination, poor light utilization, and low catalytic efficiency. Therefore, Liao et al.^16^ successfully synthesized rod-like MIL-88B-Fe mixed with g-C_3_N_4_. The presence of the heterojunction can effectively reduce the electron and hole recombination, demonstrating excellent photocatalytic performance with remarkable stability. It activated PMS and catalytic degradation of low-concentration RhB in about 1 h while maintaining good cyclic performance. Nevertheless, these catalysts can inevitably release metal ions during the reaction process and cause secondary pollution in water.

Therefore, carbon-based catalysts, such as carbon nanotubes^17^, g-C_3_N_4_^18,19^, graphene^20^, and other metal-free PMS activators^21^, have been designed.

Photocatalytic of metal-free organic polymer driven by visible light is considered a promising method for PMS activation due to its stability, mild reaction conditions, good solar energy utilization, and high efficiency^22^. However, due to ballistic electron scattering, carbon-based catalyst materials, such as graphene, g-C3N4, and carbon nanotubes, exhibit high electrical conductivity. Hence, they suffer from poor charge separation with a high recombination rate^23,24^. To tackle this issue, doping or introducing surface defects are typically employed^25^. However, an excess of defects can hinder charge separation and result in a high density of structural defects. Furthermore, adjusting the electronic structure of these materials is also limited^26^ due to the rigidity and chemical inertness of the carbon chemicals.

A covalent organic framework (COF) is an emerging material with a controllable crystal structure formed by stable periodic aromatic units through covalent bonds^27^. Two-dimensional COFs have excellent photocatalytic ability because of their extended Sp^2^ hybridized orbit structure, which can promote charge carrier transport^28^. More importantly, the presence of electron donors and acceptors in the same COF is essential for stabilizing the photoexcited electrons and holes readily used for redox reaction to activate PMS. Recently, Yang et al.^29^ found that COF-PRD synthesized from Tp and pyridine can photocatalytically activate PMS to degrade bisphenol A (BPA). TpTt-COF is a novel covalent organic framework material with a broad spectral response and effective light absorption in the visible range. It is synthesized through the Schiff base polymerization of Tp (phloroglucinol, containing -CHO groups) and Tt (melamine, containing -NH_2_ groups). Bhadra et al.^30^ synthesized TpTt-COF with a wide range of substrate scope for visible light excitation. In the presence of blue LED, TpTt-COFs were excited from the ground state to the singlet state, resulting in a more energy-stable trilinear state and interacting with trans-stilbene to form the excited states. This energy transfer aids trans-stilbene to convert to cis-stilbene through a di-radical intermediate. Feng et al.^31^ demonstrated that TpTt-COF is stable and versatile in oxidative coupling of amine, such as Aniline, to air driven by visible light. Furthermore, TpTt-COF is highly stable^32^ and can be easily recovered from the catalytic system, enabling efficient reuse. Unlike materials such as graphene, TpTt's electronic structure can be modified by introducing auxiliary functional groups^33,34^. Hence, their photocatalytic potential can be chemically optimized.

This study marks a pioneering exploration of the combined application of TpTt-COF and PMS for the visible light degradation of organic dyes. The synthesized orange TpTt-COF exhibits superior crystallinity compared to some other COFs^35^ and shows broad visible light absorption at a wavelength of 708 nm. Additionally, it demonstrates outstanding thermal stability, reaching up to 200 °C. This TpTt-COF demonstrates robust stability in acidic and alkaline water environments^36^. The creative combination with PMS makes it applicable in the field of photodegradation of organic dyes. Compared to popular transition metal photocatalysts such as Fe-MOF^37^, the process of purifying organic dyes may lead to secondary heavy metal contamination. The activation of PMS by non-metallic photocatalysts under visible light is environmentally friendly. TpTt-COF exhibits a degradation rate 13.9 times higher than PMS-combined g-C_3_N_4_^38^ and 1.6 times higher than TpTt-COF alone under identical conditions is attributed to its surface electron donor and acceptor, enabling simultaneous activation of PMS and the generation of a synergistic effect. Crucially, even after five consecutive degradation cycles, TpTt-COF maintains an impressive 83.2% degradation efficiency and stability, highlighting its excellent potential in the field of water environmental degradation. This study not only introduces a novel and highly efficient photocatalytic system mediated by PMS but also contributes valuable insights into the mechanisms governing photocatalysis for the degradation of organic pollutants in water environments.

## Experimental

### Chemicals and materials

All chemicals are AR grade. Anhydrous ethanol, Methyl Orange (MO), Rhodamine B (RhB), Methylene Blue (MB), 1,3,5-triformylphloroglucinol (${\text{Tp}}$), and melamine (Tt) were obtained from Shanghai Titan Technology Co., Ltd. Acetic acid (HAc) dimethyl sulfoxide (DMSO), *N,N*-dimethylacetamide (DMAc), potassium hydrogen monopersulfate (PMS), methanol, and Nafion perfluorinated resin were obtained from Shanghai Macklin Biochemical Co., Ltd. TiO_2_ nanoparticles (P_25_, ~ 80% of anatase and ~ 20% of rutile) were obtained from Degussa (Evonik, Germany). Deionized water (DI water, ER = 18.5 MΩ·cm^−1^) was prepared in our laboratory.

### Synthesis of TpTt-COF

**Figure 1:**
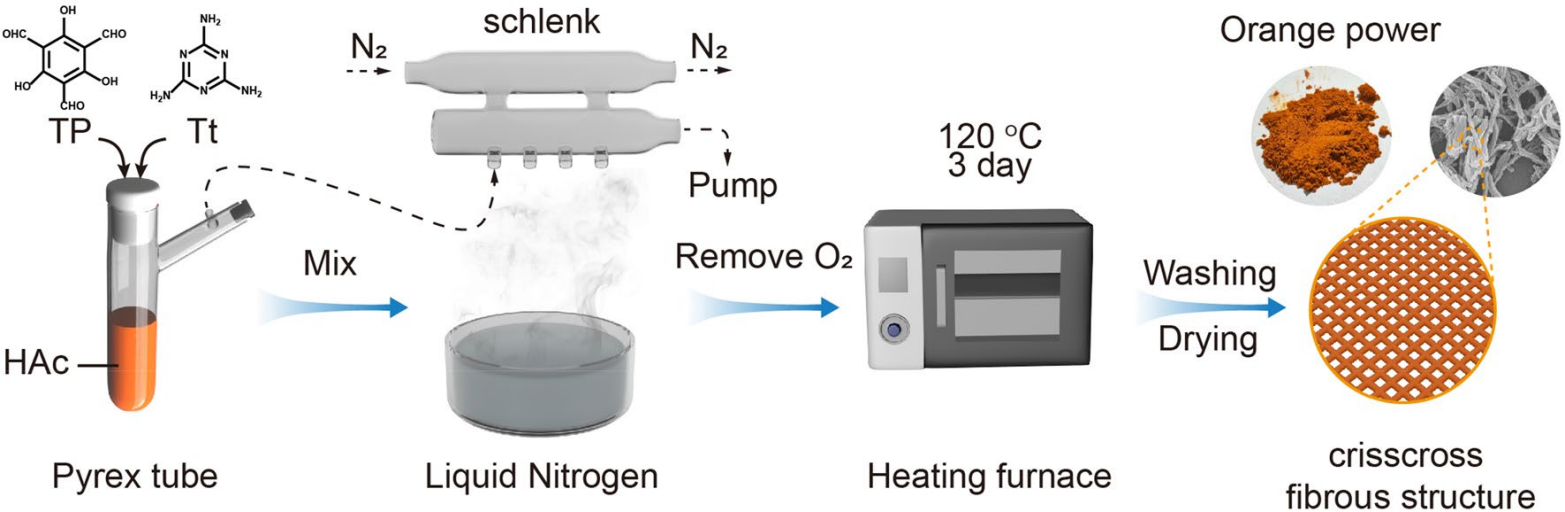
Schematic diagram of the preparation process for TpTt-COF photocatalyst.

As shown in Fig. 1, a mixture of ${\text{Tp}}$ (189 mg) and ${\text{Tt}}$ (60 mg) was dissolved in a mixed solvent containing 3 mL of DMAc, 6 mL of DMSO, and 0.9 mL HAc (6 M) in a Pyrex tube (35 mL). The solution was degassed through three freeze–pump–thaw cycles before being sealed and heated at 120 °C for three days. After cooling down, the solid product was separated by centrifugation and washed with DMAc and acetone three times. The dark orange-colored TpTt-COF powder was collected after being dried at 80 °C 12 h.

### Synthesis of standard reference materials

Choosing the most common photocatalytic materials, Titanium dioxide (TiO_2_, purchased in the market) containing transition metals, and the metal-free carbon-based material g-C_3_N_4_ as the control. The synthesis process of g-C_3_N_4_ is as follows: a typical procedure, 5 g of melamine was heated at 500 °C for 2 h with a heating rate of 20 °C/min in air. Then heated at 520℃ for 2 h. After cooling to room temperature, g-C_3_N_4_ in canary yellow was obtained.

### Characterization

The crystal structures were identified by Powder X-ray diffraction (PXRD, Rigaku Smartlab) with Cu Kα (1.5418 Å) radiation over the 2θ range of 5^°^–40^°^ and at a scanning step of 0.02^°^. The morphologies of the samples were observed by a scanning electron microscope (SEM, Hitachi SU8010). Fourier transform infrared (FTIR) spectra were collected from KBr pellets on a Spectrum One FTIR spectrophotometer (Perkin-Elmer, USA) from 4000 to 500 cm^−1^ at a resolution of 2 cm^−1^. The thermal stability was investigated by a TG-DTG 6300 thermogravimetric analyzer (EXSTAR, Japan) from room temperature to 800 ℃ at a heating rate of 10 °C/min in N_2_ flow. The band gap was obtained by UV–vis diffuse reflectance spectroscopy (UV–vis DRS, Shimadzu, Japan), collected from a dry-pressed disc sample on a PerkinElmer Lambda 650S spectrophotometer with BaSO_4_ as the reference. The specific surface area was determined by the Brunauer–Emmett–Teller (BET) method, and the pore size distribution was analyzed by the Barrett-Joyner-Halenda (BJH) model. The dye concentrations were monitored by a UV–vis spectrometer (752 N, INESA, Shanghai). The elemental valence structures were analyzed by X-ray photoelectron spectroscopy (XPS, ESCALAB 250Xi, Thermo Fisher Scientific, USA). The C1s peak at 284.8 eV was used to calibrate binding energies. The photoelectrochemical current and electrochemical impedance spectroscopy measurements were conducted using an electrochemical workstation (CHI760E, Shanghai Chenhua, China).

### Analytic method for determining dyes concentrations

The Methyl Orange (MO), Rhodamine B (RhB), and Methylene Blue (MB) solutions were sampled by transferring a small volume of the solution and centrifuging to remove TpTt solid. Their concentrations were analyzed by a UV–vis spectrophotometer at the peak absorption wavelengths of 463 nm, 554 nm, and 664 nm, respectively.

### Photocatalytic experiments

The photocatalytic activity of the TpTt-COF was assessed by degrading different dyes (RhB, MO, and MB) under simulated visible light irradiation with PMS. A Xenon arc lamp (CHF-XM-500 W, Perfectlight, China) equipped with a UV cutoff filter (λ ≥ 400 nm) was used as the excitation source. An optical power meter (FZ400, NBeT, China) was used to ensure the optical density of 100 mW/cm^2^, corresponding to the solar energy on Earth. Based on previous research^39,40^ and incorporating the insights from Figs. 5d, 6c, d, improvements were made to the method of photocatalytic degradation of organic dyes. 5 mg photocatalyst powder was uniformly dispersed in 110 mL dye aqueous solution with an initial dye concentration of 10 mg/L at pH 3. To reach the adsorption–desorption equilibrium of the dyes, the mixture was stirred in the dark for 30 min before adding 25 mg of PMS and starting the photoexcitation. The processed dye solution of 4 mL was collected within the designated time interval, followed by centrifuging before measuring the concentrations. Each measurement was performed three times.

### Electrochemistry measurements

Electrochemical analysis was performed using a conventional three-electrode system. Generally, catalyst-coated ITO glass (1 cm × 2 cm × 1.1 mm) was used as the working electrode. An Ag/AgCl electrode was used as the reference electrode, and a platinum foil was used as the counter electrode. The ITO substrate was ultrasonically cleaned in deionized water, acetone, and ethanol for 1 h each and then naturally dried. 10 mg catalyst powder was mixed with 1 mL ethanol and 35 μL adhesive (Nafion perfluorinated resin). The resulting liquid was added dropwise on the dried ITO glass 3 times with each drop volume of 30 μL. After the glass was naturally air-dried, the electrode was immersed in the electrochemical cell, containing 20 mL Na_2_SO_4_ solution (0.2 M) as the electrolyte. Transient photocurrent and electrochemical impedance spectroscopy measurements were conducted under illumination, while Mott-Schottky analysis was performed in the dark. The photoelectrochemical measurements were carried out using a CHI760E workstation (Shanghai Chenhua Instrument) illuminated by a 300 W xenon lamp (CELHXF300) equipped with a UV cutoff filter (λ ≥ 400 nm). Illumination power was calibrated to be 100 mW/cm^2^ using a FZ400 optical power meter.

## Results and discussion

### Characterization

**Figure 2:**
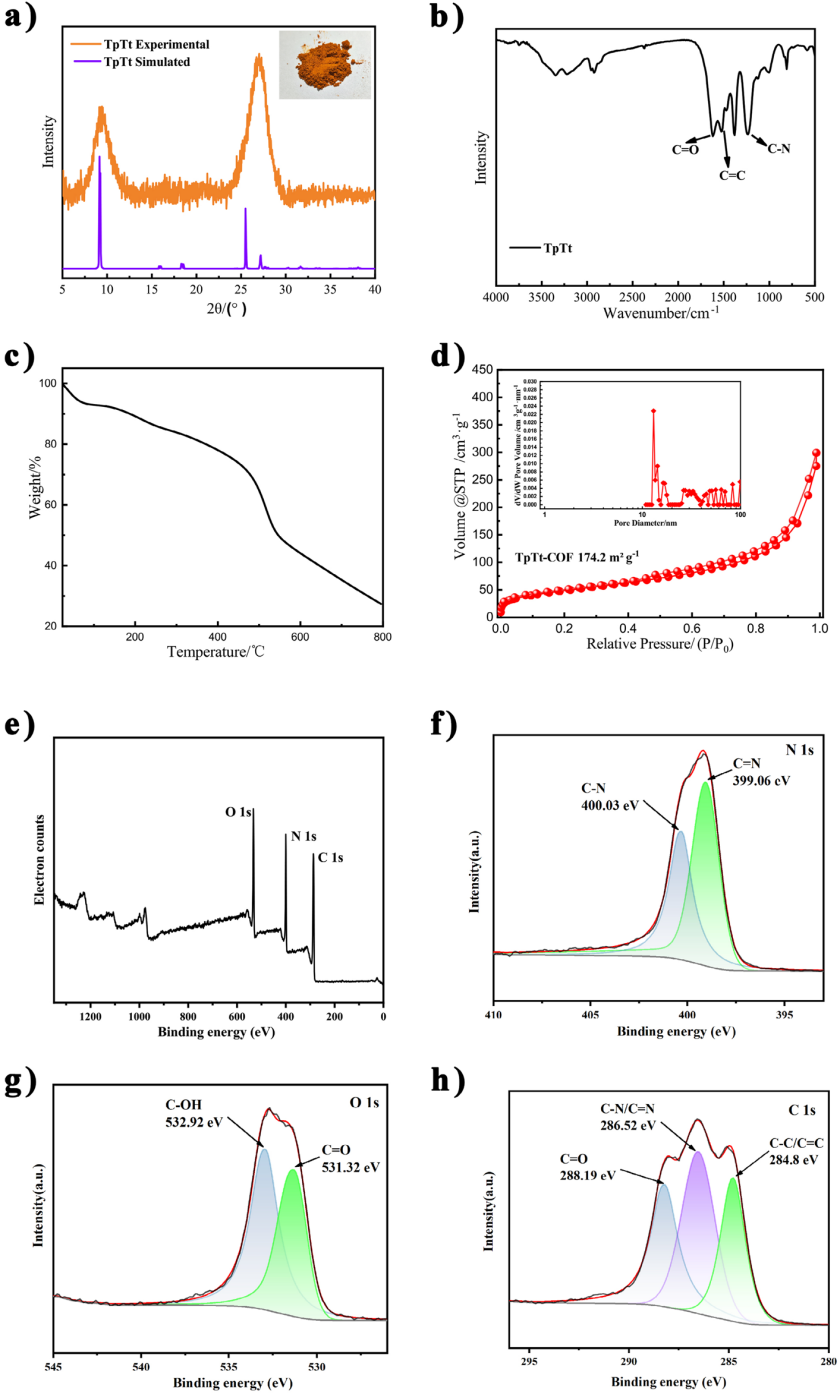
(**a**) The measured and simulated XRD patterns, (**b**) FTIR spectrum, (**c**) TGA data, (**d**) N_2_ adsorption–desorption isotherms of TpTt-COF, the inset in (**a**) shows the color photograph of the sample, and the inset in (**d**) is the pore diameter distribution from the BJH analysis. (**e**) XPS spectrum of TpTt-COF. High-resolution XPS spectra of (**f**) N 1s, (**g**) O 1s, (**h**) C 1s of TpTt-COF.

The crystal structure of TpTt-COF was investigated using PXRD, as shown in Fig. 2a. Distinct peaks at 9.7° and 27.4° were observed, attributed to the diffraction from the (100) and (002) planes, respectively. The presence of these peaks signifies the successful formation of crystallized TpTt-COF^30^. The corresponding peak shape is consistent with previous studies and simulated XRD. The chemical structure of COFs was analyzed by FTIR, as illustrated in Fig. 2b. The peak at 1620 cm^−1^ is attributed to the stretching vibration of the carbonyl group C=O in the β-ketoenamine moiety. The peaks at 1523 cm^−1^ and 1236 cm^−1^ correspond to the stretching vibrations of the C=C enamine carbon and the C–N bond at 1236 cm^−1^, respectively. The peaks at 1384 cm^−1^ and around the other fingerprint regions originate from the monomers of Tp and Tt^36,41^, respectively, suggesting the formation of β-keto groups within the TpTt-COF. The XRD and FTIR data confirm the successful synthesis of TpTt-COF. Thermogravimetric analysis (TGA) was utilized to assess the thermal stability of COFs. As illustrated in Fig. 2c, a slight weight reduction was noticeable at low temperatures between 100 and 200 °C, likely due to the evaporation of water and residual organic solvents. When the temperature reaches 500 °C, the mass decreases significantly due to the total decomposition of TpTt. These observations imply that the COF structure is thermally stable under 200 °C. The Brunauer–Emmett–Teller (BET) method was used to analyze the specific surface area and pore structure of COFs. Figure 2d illustrates a type I isotherm with a BET specific surface area of 174.2 m^2^/g for the TpTt. This observation implies that TpTt-COF features a mesopore structure exceeding 10 nm in pore diameter, as shown from the BJH(Barrett-Joyner-Halenda) analysis in the inset in Fig. 2d. The pore structure agrees with the theoretical structure of TpTt-COFs. X-ray photoelectron spectroscopy (XPS) was employed to thoroughly examine the chemical composition of TpTt-COF. The XPS analysis, depicted in Fig. 2e, includes an XPS survey scan that distinctly illustrates the presence of carbon (C), nitrogen (N), and oxygen (O) elements within TpTt-COF. The N 1s XPS spectrum, presented in Fig. 2f, exhibits two prominent peaks corresponding to C–N and C=N in the triazine ring, with peak positions at 400.03 and 399.06 eV, respectively. In the O 1s XPS spectrum (Fig. 2g), the C–OH vibration peak of TpTt-COF is identified at 532.92 eV, while the C=O peak is observed at 531.32 eV. Simultaneously, the C 1s XPS spectrum (Fig. 2h) reveals three peaks at 284.8 eV, 286.52 eV, and 288.19 eV, corresponding to aromatic *sp*^*2*^ hybridization C–C/C=C, C–N/C=N of triazine, and C=O of carboxylic acid. These findings, aligned with the specific figures, furnish essential details affirming the successful synthesis of TpTt-COF^42^, seamlessly connecting with PXRD and FTIR analyses.

**Figure 3:**
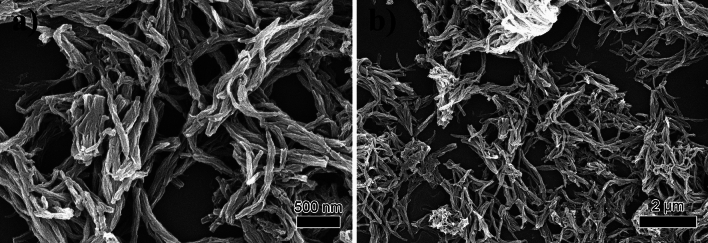
(**a**) High and (**b**) low magnification SEM images of TpTt-COF.

The SEM morphology of the TpTt-COF sample is shown in Fig. 3a, b. TpTt-COF manifests as a crisscross fibrous structure with an average diameter of roughly 90 nm and an extending length of several micrometers.

**Figure 4:**
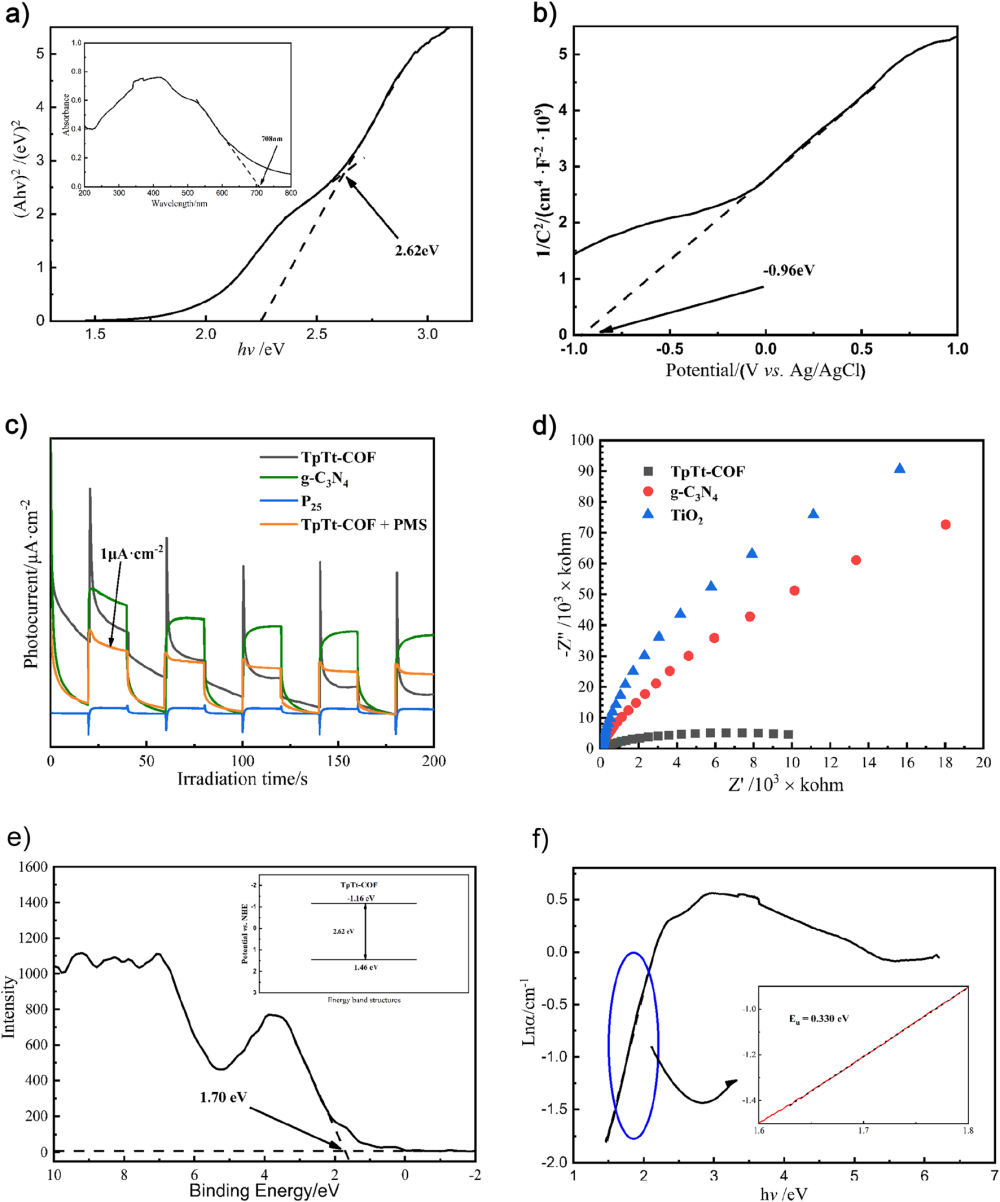
(**a**) The Tauc plot and UV–vis diffuse reflectance absorption spectrum (inset), (**b**) Mott–Schottky analysis, (**c**) transient photocurrent responses, (**d**) EIS Nyquist plots, (**e**) XPS valence band spectra and the energy-band structure (inset), (**f**) Urbach tail analysis of TpTt-COF.

The optical characteristics of COFs were investigated with UV–vis DRS. The inset in Fig. 4a shows the absorption spectrum of TpTt in the visible region with three peak maxima at 350, 420, and 525 nm. TpTt-COF offers a remarkably broad spectral response implying an extensive light-harvesting capacity, attributed to its extensive π-electron delocalization and the rapid occurrence of intramolecular charge transfer. The light absorption spectrum agrees with the dark orange color of TpTt shown in Fig. 2a, allowing its efficient absorption of a substantial portion of blue-green light, leading to a remarkably high efficiency in solar light utilization. Furthermore, the Tauc plot, as shown in Fig. 4a, was derived from the Kubelka–Munk equation with the bandgap energy of 2.62 eV for TpTt^43–45^, similar to the theoretical value of 2.657 eV^30^.

### Photoelectrochemical analysis

Mott-Schottky analysis was employed to obtain the flat-band potential. As illustrated in Fig. 4b, TpTt-COF exhibits positive slopes in the Mott–Schottky plot, indicating an n-type semiconductor. The flat-band potential (E_fb_) of TpTt-COF was −0.96 V vs Ag/AgCl. It's widely recognized that the flat-band potential of an n-type semiconductor is close to its conduction band (CB) potential. Hence, the E_CB_ of TpTt is approximately −1.16 V vs. normal hydrogen electrode (NHE). From the band gap energy of 2.62 eV, the valence band (VB) potential of TpTt-COF is 1.46 V vs. NHE.

Figure 4e presents the XPS spectrum showing the valence band edge of TpTt at 1.70 V with the instrument's work function (E_function_) of 4.2 eV. It can be obtained by the formula ^43^ (E_VB,NHE_ = E_function_ + E_VB,XPS_ − 4.44 ) This concurs with the previously inferred valence band value of TpTt, resting at 1.46 V. In the inset in Fig. 4e, the band structure is presented. Figure 4f shows the Urbach tail analysis of the extinction coefficient, which is related to the density of defects^46–48^. Eu = 0.33 eV indicates mixed phases or some defects in TpTt. This particular characteristic is also evident in Fig. 4a, where a marginally smaller slope is observed in addition to the primary slope, affecting the Urbach tail with additional adsorption peak at 420nm^43,49^.

Transient photocurrent response was analyzed to investigate the photogenerated charge transfer. Referring to previous studies^50^, g-C_3_N_4_ and TiO_2_ were employed as conventional photocatalysts for a comparative analysis of the Photoelectrochemical properties and Photocatalytic activity of TpTt-COF. This approach enhances the objectivity of the experimental results. Figure 4c illustrates that TpTt-COF, g-C_3_N_4,_ and TiO_2_ exhibited rapid on–off cycle photocurrent responses when exposed to visible light. This behavior is directly linked to the effectiveness of charge separation. Notably, the TpTt electrode displayed a distinctive initial spike in current^51^, which was insignificant for the g-C_3_N_4_ and TiO_2_. We believe this is directly associated with the unique electronic structure of TpTt containing alternating adjacent electron donors and acceptors. When visible light irradiated the TpTt surface, photoexcited electrons were collected by the ITO and transferred to the counter electrode. Meanwhile, the excited holes were attracted to the electron donors (Tt) accumulated on the TpTt surface, leading to a positively charged surface. Subsequently, the accumulated holes will attract the photoexcited electrons on the (Tp) sites, initiating competition for electron migration. As a result, the photocurrent intensity decreased exponentially until the balance of surface charge density was achieved. Figure 4 reveals that g-C_3_N_4_ exhibits the highest photocurrent, whereas TpTt experiences a substantially lower current intensity due to the charge trapping and recombination. However, introducing an appropriate amount of PMS significantly recovered the photocurrent intensity, while the initial peak current weakened considerably. This observation suggests that PMS can react with both the photoexcited electrons and holes directly on the surface of the TpTt.

EIS measurements were carried out to assess the charge transfer resistance at the electrode/electrolyte interface and examine charge transfer within COFs. As depicted in Fig. 4d, the semicircle diameter of the Nyquist plot from the TpTt-COF is markedly smaller than that from g-C_3_N_4_ and TiO_2_, indicating a lower charge transfer resistance, possibly due to the presence of both electron donor and acceptor at the adjacent part of the TpTt molecule.

### Photocatalytic activity and mechanism

**Figure 5:**
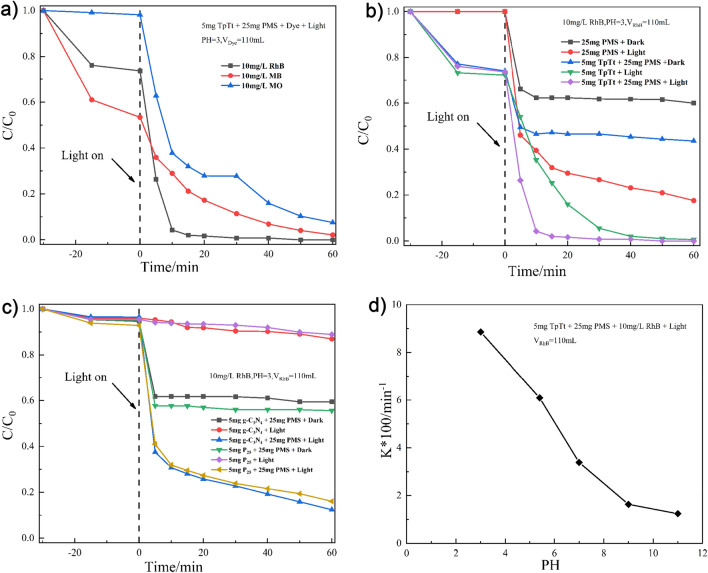
(**a**) The concentration evolution during the photocatalytic degradation of RhB, MO, and MB dyes with PMS and TpTt-COF at pH3, (**b**) RhB degradation with PMS, TpTt, and their combination in dark and visible light excitation, (**c**) g-C_3_N_4_ and P_25_(TiO_2_) control group, (**d**) photocatalytic RhB degradation rate constant as a function of pH.

To evaluate the photocatalytic efficacy of TpTt-COF in conjunction with PMS, dye degradation kinetics measurements were carried out for solutions containing 10 mg/L of RhB, MB, and MO, as illustrated in Fig. 5a. While TpTt-COF demonstrated the highest adsorption capacity for MB during the initial adsorption process in the dark, the subsequent photocatalytic process revealed that RhB exhibited the highest photodegradation rate. Hence, RhB was selected as an example for further investigation into TpTt-COF's performance in degrading dyes.

Figure 5b shows that PMS alone exhibited a low degradation rate in both dark and illuminated conditions with the rate constants of 0.655 min^−1^ and 2.234 min^−1^, respectively. For the TpTt without PMS, a degradation rate constant of 5.502 min^−1^ was achieved. However, with TpTt combined with PMS, it was increased to 8.856 min^−1^, 1.6 times that of TpTt alone or 4.0 times that of PMS alone. Hence, the fast degradation kinetics can only be achieved with the synergistic effects of TpTt and PMS. In comparison, from Fig. 5c, the degradation rate constants for g-C_3_N_4_ and P_25_(TiO_2_) combined with PMS are 0.636 min^−1^ and 0.732 min^−1^, respectively. However, without PMS, the corresponding K values decrease to 0.146 min^−1^ and 0.112 min^−1^, respectively. Thus, TpTt offers much better photocatalytic performance through the activation of PMS in comparison with traditional or commercial photocatalysts, such as g-C_3_N_4_ and P_25_(TiO_2_). This is because TpTt can supply both photoexcited electrons and holes to facilitate the activation of PMS and generation of ·O_2_^-^and ·SO_4_^-^ radicals. For traditional n-type photocatalysts, normally only excited holes are responsible for producing oxidative radicals, and the photoexcited electrons are consumed by producing hydrogen on the counter electrode in an aquatic environment. Figure 5d illustrates that the TpTt's photocatalytic performance gradually improves as the pH decreases. This improvement may be attributed to the nature of surface functional groups on the catalyst^52^, which might affect the balance between the trapped photoexcited electrons and holes.

**Figure 6:**
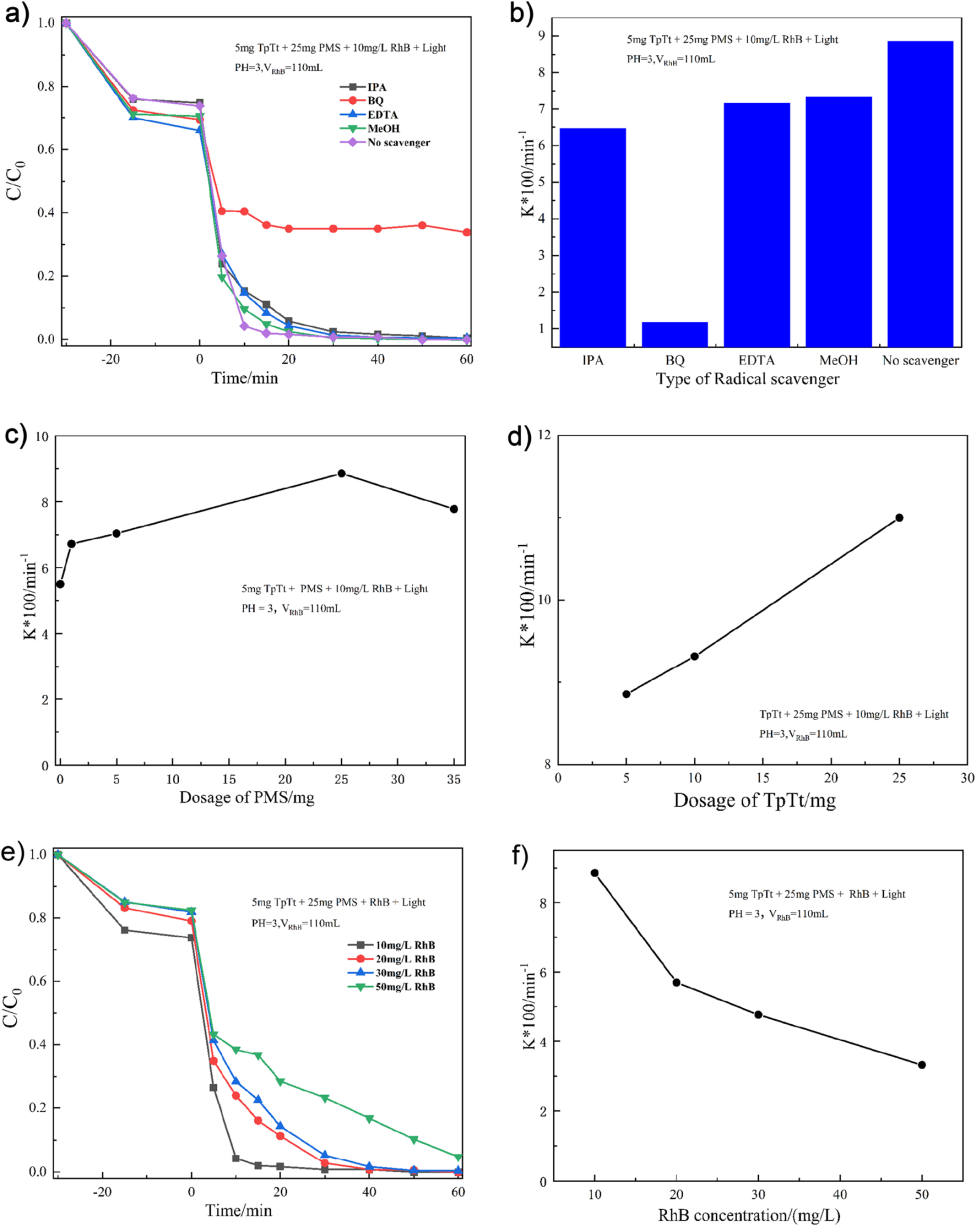
(**a**) The RhB degradation kinetics with added radical scavengers, (**b**) the measured rate constants with different radical scavengers, (**c**) the effects of PMS dosage on the rate constant, (**d**) the effects of TpTt dosage on the rate constant, (**e**) the RhB degradation kinetics as a function of initial RhB concentration, (**f**) the change of degradation rate constant as a function of initial RhB concentration.

To investigate the synergistic mechanism for the photocatalytic degradation of dyes using TpTt-COF enhanced with PMS, different radical scavengers, including IPA, BQ, EDTA, and MeOH, were added to quench ·OH, ·O_2_^−^, h^+^, and ·SO_4_^−^, respectively. Figure 6a shows that the addition of IPA, EDTA, and MeOH had similar minor inhibitory effects, indicating that ·OH, h^+^, and ·SO_4_^−^ play less important roles in the degradation of RhB. However, introducing an appropriate amount of BQ exhibited the strongest inhibitory effect by decreasing the rate constant by 87%, indicating that ·O_2_^−^ is the dominant active radical. The result is summarized in Fig. 6b. Furthermore, Fig. 6c reveals that the K value increases with increasing PMS mass, reaching a maximum value with 25 mg of PMS. Hence, excess PMS can lead to the generation of ·OH and ·SO_4_^-^, reducing the concentration of ·O_2_^−^ with decreased performance.

1 $$ {\text{TpTt-COF}} + {\text{ h}}v \to {\text{h}}^{ + } + e^{ - } $$

2 $$ e^{ - } + {\text{ O}}_{{2}} \to \cdot{\text{O}}_{{2}}^{{ - }{}} $$

3 $$ {\text{h}}^{{ + }{}} + \cdot{\text{O}}_{{2}}^{ - } \to^{{1}} {\text{O}}_{{2}} $$

4 $$ e^{ - } + {\text{ HSO}}_{{5}}^{ - } + {\text{H}}^{ + } \to \cdot{\text{SO}}_{{4}}^{ - } + {\text{ H}}_{{2}} {\text{O}} $$

5 $$ \cdot{\text{SO}}_{{4}}^{ - } + {\text{ H}}_{{2}} {\text{O }} \to {\text{ HSO}}_{{4}}^{ - } + \, \cdot{\text{OH}} $$

The excitation process is proposed here. When light illuminates the surface of TpTt, it initiates process (Eq. 1) to produce excited electrons and holes. The reaction of electrons with O_2_ produces the ·O_2_^−^ (Eq. 2), which can recombine with hole (Eq. 3), causing a rapid decline in photocurrent intensity and the appearance of the initial photocurrent spike^29,53–55^. Nevertheless, introducing PMS significantly enhances the performance, increasing it by 60%. This improvement can be attributed to:

6 $$ {\text{HSO}}_{{5}}^{ - } + {\text{h}}^{ + } \to \, \cdot{\text{SO}}_{{5}}^{ - } + {\text{H}}^{ + } $$

7 $$ \cdot{\text{SO}}_{{5}}^{ - } + {\text{ H}}_{{2}} {\text{O }} \to {\text{ H}}_{{2}} {\text{SO}}_{{4}} + \cdot{\text{O}}_{{2}}^{{ - }{}} $$

Upon introducing PMS, the photogenerated electrons and holes will activate PMS, forming ·SO_4_^−^ (Eq. 4) and ·SO_5_^−^ (Eq. 6) radicals, respectively. The ·SO_4_^−^ will react with H_2_O to produce the oxidative radical of ·OH (Eq. 5), quenched by IPA (Fig. 6b). Meanwhile, the SO_5_^−^ will also react with H_2_O, producing the reactive ·O_2_^−^ radical (Eq. 7)^56^. Hence, two possible pathways exist for producing the ·O_2_^−^ radicals through either the direct reduction of O_2_ by photoexcited electrons on TpTt (Eq. 2) or the oxidation of H_2_O mediated by activated PMS (Eqs. 6 and 7). Nonetheless, adding PMS will utilize both photoexcited electrons and holes in the degradation of RhB with a synergistic effect. Figure 6d confirms that an increase in the quantity of TpTt substantially accelerated the degrading kinetics since more PMS can be activated to produce ·O_2_^−^ radicals. PMS is gradually consumed in this process since the increase in the RhB concentration confirmed the limited degradation capacity in Fig. 6e, although the initial degradation rate was maintained. After PMS was depleted, the degradation rate was significantly reduced, represented by the declining rate constant, as shown in Fig. 6f.

### Reusability and stability

**Figure 7:**
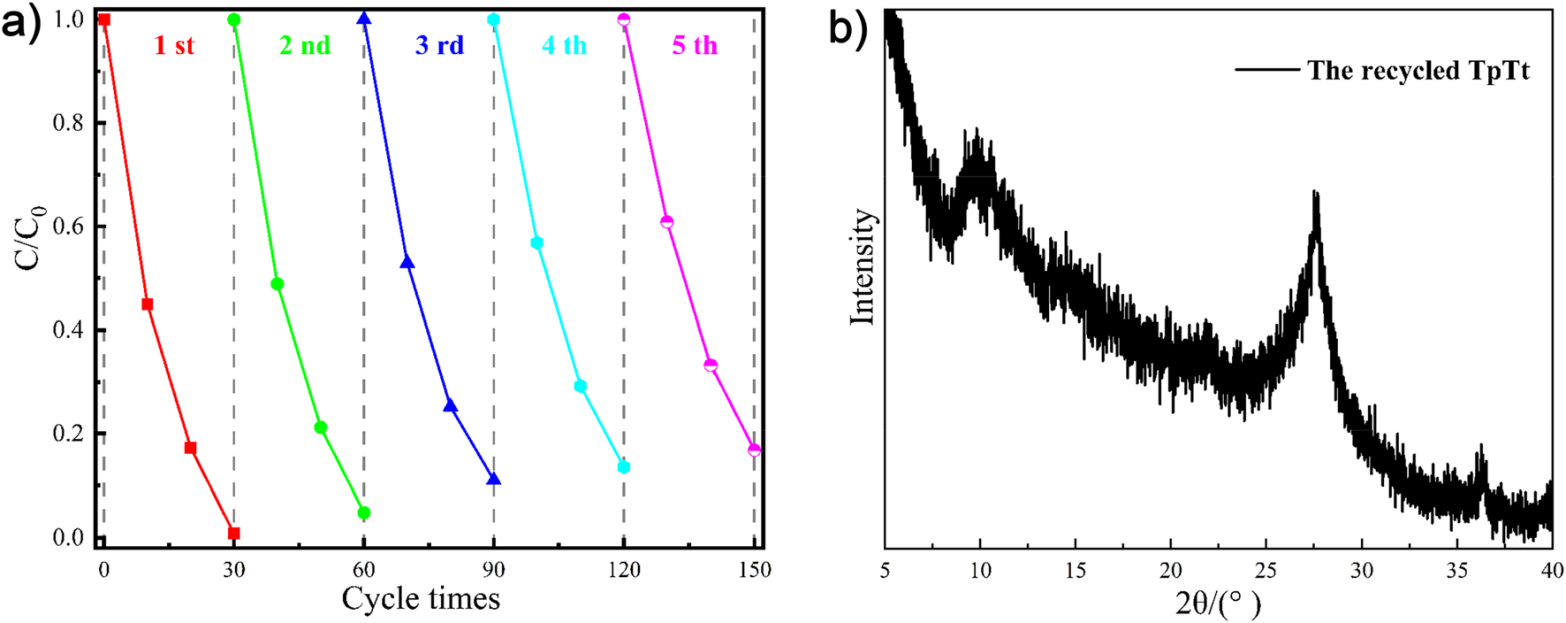
(**a**) Repeating photocatalytic degradation of RhB under visible light irradiation. (conditions: catalyst = 5 mg, PMS = 25 mg, solution volume = 110 mL and RhB = 10 mg L^−1^, PH 3), (**b**) XRD spectrum of TpTt after five cycle.

The reusability and performance stability are critical factors in assessing the commercial viability of a photocatalyst. As illustrated in Fig. 7a, even after five cycles of photocatalysis, TpTt continues to exhibit an impressive efficiency of 83.2% for RhB degradation. The XRD spectrum in Fig. 7b shows there is minimal change in the structural integrity^57,58^. Hence, the synthesized TpTt, as a reusable, efficient photocatalyst, holds considerable promise for practical applications.

**Figure 8:**
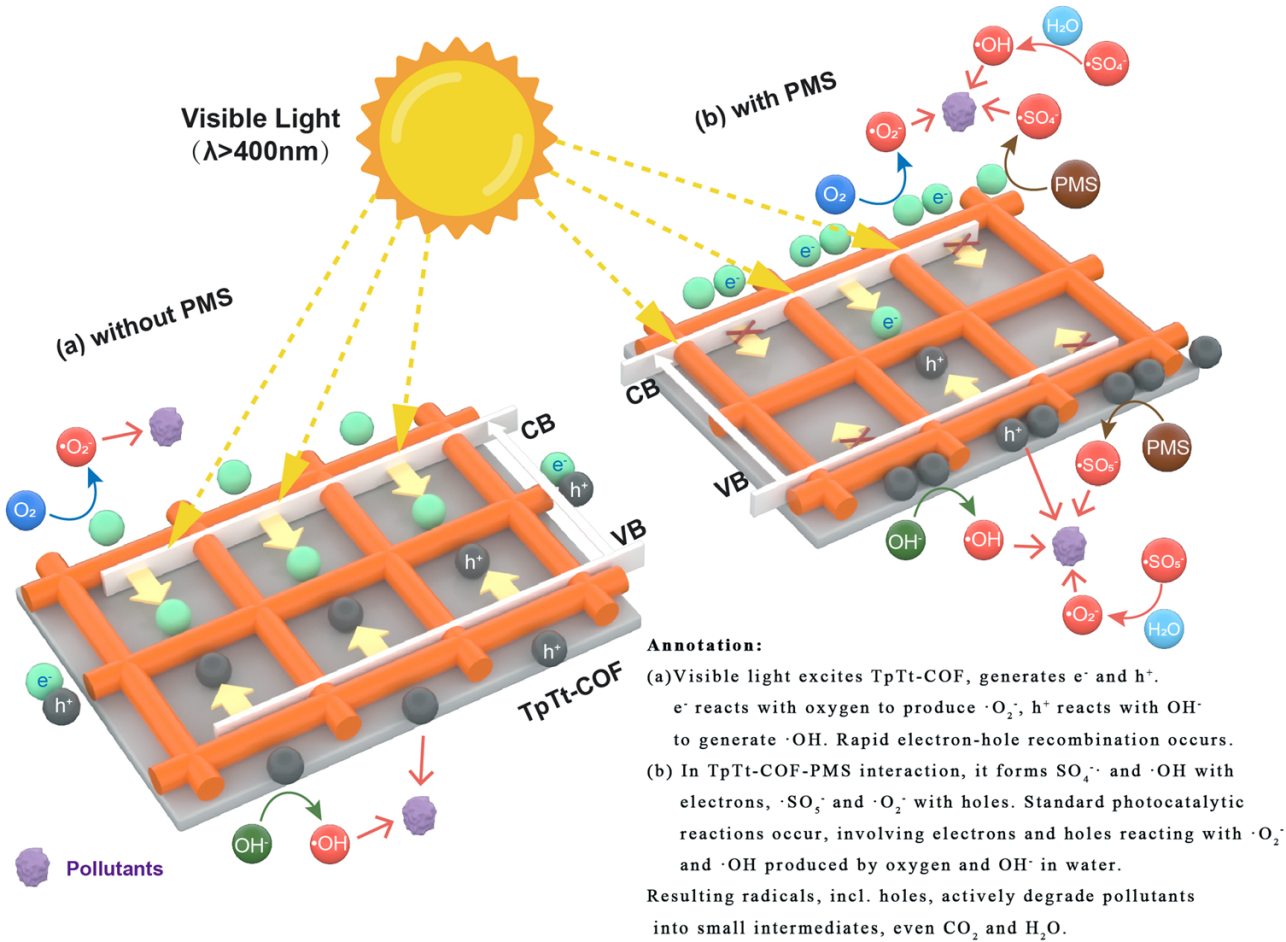
Schematic diagram of dye degradation mechanism over TpTt-COF photocatalyst (**a**) without PMS, (**b**) with PMS.

The reaction and process illustrated in Equations (1–7) are also shown in Fig. 8. Without PMS, the excitation process for the TpTt is proposed as Fig. 8a, aligning with the general mechanism of photocatalysis. When TpTt-COF is excited by visible light, electrons (e^−^) and holes (h^+^) are generated. Electrons react with oxygen in the water environment to produce ·O_2_^−^, while holes react with OH^−^ in water to generate ·OH. Of course, electrons and holes also rapidly react, representing the fast recombination of holes and electrons in TpTt-COF. The descriptions of the mechanisms mentioned above, in which TpTt is combined with PMS, can all be visually demonstrated in Fig. 8b. In the process of TpTt-COF binding with PMS, it not only reacts with electrons to produce SO_4_^−^· and ·OH but also reacts with holes to generate ·SO_5_^−^ and ·O_2_^−^. This process not only promotes the generation of various free radicals but also facilitates the effective separation of electrons and holes, significantly reducing the recombination of electrons and holes, presenting an intriguing synergistic effect. Of course, in the case of combining with PMS, reactions conforming to the fundamental photocatalysis rules also occur, where electrons and holes react with ·O_2_^−^ and ·OH produced by oxygen in the water environment and OH^−^ in water, respectively. The generated free radicals, including holes themselves, actively participate in the degradation process of pollutants, breaking them down into small molecular intermediates, and even into carbon dioxide and water.

## Conclusions

This study achieves innovative degradation of organic dyes under visible light by exploring the combined application of TpTt-COF and PMS. The synthesized orange TpTt-COF monomer demonstrates outstanding photocatalytic properties, environmental adaptability, and sustainability, positioning itself as a potential candidate for the photocatalytic degradation of organic dyes in water environments. The study effectively addresses the challenge of high charge recombination in TpTt-COF monomers. In comparison to traditional photocatalytic materials, the degradation rate of TpTt-COF combined with PMS is 13.9 times higher than PMS combined with g-C_3_N_4_ and 1.6 times higher than TpTt-COF alone under identical conditions. The research delves into the underlying mechanisms of this degradation process. Under visible light excitation, TpTt-COF, when combined with PMS, engages in reactions with electrons (e^−^) to produce SO_4_^−^· and ·OH, and with holes (h^+^) to generate ·SO_5_^−^ and ·O_2_^−^. This dual reaction mechanism enhances the generation of various free radicals and ensures the effective separation of e^−^ and h^+^, resulting in a remarkable synergistic effect. Radical quenching experiments validate the crucial role of O_2_^−^· radicals, while the formation of ·OH and SO_4_^−^· radicals further intensifies the degradation.

Crucially, even after five consecutive degradation cycles, TpTt-COF maintains an impressive 83.2% degradation efficiency and stability. This study not only introduces a novel and highly efficient photocatalytic system mediated by PMS, but also provides valuable insights into the mechanisms governing the degradation of organic pollutants in water environments. These findings contribute to the practical application of photocatalytic technology and offer beneficial guidance for environmental management and pollution control.

## Data Availability

The datasets generated during and/or analysed during the current study are available from the corresponding author on reasonable request.
